# Reduced influenza viral neutralizing activity of natural human trimers of surfactant protein D

**DOI:** 10.1186/1465-9921-8-9

**Published:** 2007-02-05

**Authors:** Kevan L Hartshorn, Mitchell R White, Tesfaldet Tecle, Ida Tornoe, Grith L Sorensen, Erika C Crouch, Uffe Holmskov

**Affiliations:** 1 Boston University School of Medicine, Department of Medicine, Boston MA, USA; 2 Washington University School of Medicine, Department of Pathology and Immunology, St. Louis, MO, USA; 3 Medical Biotechnology Center, University of Southern Denmark, Odense, Denmark

## Abstract

**Background:**

Surfactant protein D (SP-D) plays important roles in innate host defense against influenza A virus (IAV) infection. Common human polymorphisms of SP-D have been found in many human populations and associated with increased risk of certain infections. We recently reported that the Thr/Thr 11 form of SP-D is associated with low serum levels and assembles predominantly as trimers as opposed to the more common multimeric forms of SP-D.

**Methods:**

Preliminary experiments were done to establish the effects of different monoclonal antibodies against SP-D on ability of SP-D to bind to or neutralize the virus. We then purified natural human trimeric and multimeric forms of SP-D from amniotic fluid and tested ability of these preparations to bind to IAV, to inhibit infectivity and hemagglutination activity of IAV in vitro.

**Results:**

In initial experiments mAbs directed against different areas on the CRD of SP-D were found to have differing effects on antiviral activity. Using an mAb that did not interfere with antiviral activity of SP-D, we confirm that natural SP-D trimers had reduced ability to bind to IAV. In addition, the trimers had reduced ability to neutralize IAV as compared to natural human SP-D multimers as well as reduced hemagglutination inhibiting activity against several strains of IAV. Natural SP-D trimers also had different interactions with human neutrophil peptide defensins (HNPs) in viral neutralization assays as compared to multimeric SP-D.

**Conclusion:**

These studies indicate that a common human polymorphic form of SP-D may modulate host defense against IAV and give impetus to clinical studies correlating this genotype with risk for IAV infection in susceptible groups. We also show that mAbs directed against different areas on the carbohydrate recognition domain of SP-D can be useful for dissecting out different functional properties of the protein.

## Background

IAV infections are a major cause of morbidity and mortality, causing ~40,000 deaths per year in the United States [1]. Recent episodes of transmission of avian IAV infection to humans underscore the ongoing potential for pandemic caused by IAV [2]. Innate immune mechanisms provide important protection against IAV in the naïve host. Among the innate immune proteins with significant anti-IAV activity are Type I interferons, tumor necrosis factor, the collectins, and defensins [3-6]. Surfactant protein D (SP-D) has particularly important roles in restricting IAV replication and limiting the severity of inflammatory responses during the first several days of infection [7-13].

SP-D has strong antiviral activity and mediates several other important functions some of which may be important during infection, including maintenance of surfactant homeostasis in the lung, clearance of apoptotic cells, enhancement or inhibition of uptake of various organisms by phagocytes, and inhibition of inflammatory reactions in the lung [8,14-19]. SP-D could also contribute to adaptive immune responses through facilitating presentation of antigen by dendritic cells [20] and decreasing lymphocyte activation and proliferation [21]. SP-D has also been shown to bind to a few host molecules including scavenger receptor rich glycoprotein 340 (gp-340), CD14 [22], microfibril-associated protein 4 (MFAP4), decorin, DNA and human neutrophil defensins (HNPs) [23-28]. Gp-340 and HNPs have significant anti-influenza activity in their own right and have complex interactions with SP-D in various assays of antiviral activity [28-32].

SP-D is a collagenous lectin that assembles as a trimer with a globular carbohydrate recognition domain (CRD) and an extended collagenous domain. These trimers can further multimerize into assemblies of 4 or more trimers linked at the N-terminus by disulfide bonds [33]. There are several common polymorphic forms of SP-D some of which appear to be of functional and clinical significance. We have recently shown that the Thr/Thr11 form of SP-D assembles *in vivo *and in recombinant preparations predominantly as trimers, lacking the usual higher multimeric forms observed for other polymorphic forms of SP-D [34]. The Thr/Thr11 variant occurs in approximately 17% of the Danish population and is also associated with lower serum levels of SP-D. The trimeric form of SP-D had reduced binding to mannan, several types of bacteria and IAV, as compared to binding by the multimeric Met/Met11 form of SP-D. Interestingly, despite reduced binding to these microbes, the trimeric form of SP-D had equivalent binding to LPS [34] and to HNPs [28]. Binding to LPS or HNPs was not found to be mediated by the calcium-dependent lectin activity of SP-D (in contrast to binding to bacteria or IAV).

Additional studies in other populations have found a similar frequency of the Thr/Thr 11 variant of SP-D [35] suggesting that the polymorphism has been conserved in many populations. Furthermore, the Thr/Thr 11 variant has been associated with increased risk of *Mycobacterium tuberculosis *infection [36]. Conversely, the Met/Met11 form is associated with increased severity of respiratory syncytial virus (RSV) infection [37]. We have speculated that the Thr/Thr 11 form of SP-D may modify risk for severe IAV infection. In the current paper we demonstrate that natural SP-D in the trimeric form not only has reduced ability to bind IAV, but has less viral neutralizing activity, supporting the hypothesis that the Thr/Thr 11 form of SP-D may be associated with reduced innate defense against IAV.

## Methods

### Virus preparation

IAV was grown in the chorioallantoic fluid of ten day old chicken eggs and purified on a discontinuous sucrose gradient as previously described [38]. The virus was dialyzed against phosphate buffered saline (PBS) to remove sucrose, aliquoted and stored at -80°C until needed. Philippines 82/H3N2 (Phil82) and Brazil79/H1N1 (Braz79) strains were kindly provided by Dr. E. Margot Anders (University of Melbourne, Melbourne, Australia). The A/PR/8/34/H1N1 (PR-8) strain was a gift of Dr. Jon Abramson (Bowman Gray School of Medicine, Winston-Salem, NC). The HA titer of each virus preparation was determined by titration of virus samples in PBS with thoroughly washed human type O, Rh(-) red blood cells as described [38]. Post thawing the viral stocks contained ~5 × 10^8 ^plaque forming units/ml.

### SP-D and HNP preparations

Recombinant human SP-D (RhSP-D) was produced in stably transfected CHO-K1 cells as previously described [9]. The recombinant preparation used contains the Thr11 sequence in the N-terminus and could be separated into distinct fractions containing predominantly trimers, dodecamers or high molecular weight multimers as previously described [8,9]. In general the dodecameric fraction was used, except for experiments in which the activity of purified recombinant trimers and multimers were compared. Natural human SP-D was isolated from amniotic fluid as previously described [34]. A pool of amniotic fluid (n = 6) was centrifuged 4000 rpm, 4°C for 30 min and purified by maltosyl agarose affinity chromatography. SP-D eluted as two structural different forms and was collected in fractions 3, 4 ("high molecular weight SP-D) and 6, 7 ("low molecular weight SP-D"), respectively. As previously demonstrated, the high molecular weight fraction consists predominantly of dodecamers, whereas the low molecular weight fraction consists of trimers. The SP-D preparations were aliquoted and stored at -80°C and after thawing were kept at 4°C for short periods (1–2 weeks) in general.

The innate defense protein preparations used in this report were tested for degree of contamination with endotoxin using a quantitative endotoxin assay (Limulus Amebocyte Lysate; Bio-Whittaker, Walkersville, MD). The final concentrations of endotoxin in protein samples containing the highest concentrations of collectins were ~20–100 pg/ml (or 6–12 Endotoxin Units/ml using internal assay standard). HNP-1 and HNP-2 were purchased from Bachem Bioscience (King of Prussia, PA). The levels of endotoxin in the HNP preparations were 15 and 25 pg/μg of protein, respectively for HNP-1 and 2.

### mAbs against SP-D

MAbs 246-04 and 246-07 were raised against SP-D by inoculating mice with 10 μg/ml of human SP-D as previously described [39]. The 245-01 was raised against a recombinant fragment of human SP-D composed of only the neck and carbohydrate recognition domain (CRD), recognizes human SP-D by Western blot in reduced and unreduced form, and was shown to be effective for immunolocalization of SP-D in human tissues. By ELISA 246-04 and 246-07 react with full length human SP-D as well and neck plus CRD fragments; 245-01 reacts well with neck plus CRD fragments of human SP-D but not with full length human SP-D (data not shown). Using recombinant fragments of SP-D we have found that the 245-01 mAb reacts with the neck region of human SP-D. The 246-04 and 246-07 mAbs only recognize SP-D in non-reduced form and bind to distinct epitopes on the CRD. The 246-07 mAb probably binds to the primary calcium and saccharide binding region of the CRD since it blocks binding of SP-D to mannan [31]. In contrast, the 246-04 mAb binds to a distinct region of the CRD and does not block binding to mannan. The 246-04 mAb does, however, block binding of SP-D to gp-340 [31].

### Measurement of binding of SP-D to IAV

**Figure 1:**
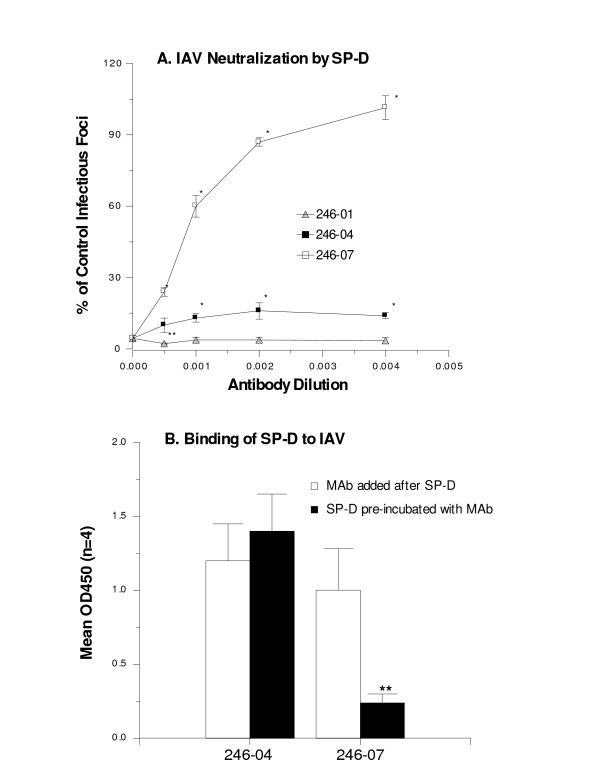
**Effect of mAbs on viral neutralizing and binding activity of SP-D**. Three mAbs directed against the CRD of SP-D were tested as indicated. In panel A, a fixed dose of recombinant SP-D multimers (100 ng/ml) was used to neutralize the Phil82 strain of IAV. This concentration of SP-D reduced the number of infectious foci to 4.4 ± 1.2% of control (shown at 0 on the y axis). Neutralization was assessed by fluorescent focus assay and expressed as % of control infectious foci in SP-D treated samples compared to control. Increasing concentrations of the mAbs were pre-incubated with SP-D prior to the neutralization assay. The 246-07 markedly inhibited neutralizing activity (* indicates p < 0.05 vs control). Although 246-04 also inhibited neutralizing activity modestly as indicated its effect was significantly less than 246-07. The 245-01 antibody did not inhibit neutralizing activity but slightly increased neutralization at one concentration (indicated by **). In panel B, the 246-07 and 246-04 mAbs were used to detect SP-D bound to Phil82 IAV by ELISA. Recombinant SP-D multimers were used at 200 ng/ml. Where indicated the antibodies were added after SP-D bound to IAV or added to SP-D prior to adding SP-D to IAV. The 246-07 mAb significantly reduced binding of SP-D when pre-incubated with SP-D prior to addition to the virus (indicated by **). Results are mean ± SEM of 4 experiments.

Binding of collectins to IAV were assessed by ELISA. Briefly, wells were incubated overnight at 4°C with a 1:250 dilution of Phil82 stock in coating buffer. Following washing with PBS, the plates were blocked with PBS containing 2.5% fatty acid and endotoxin free BSA (Sigma, fraction V, fatty acid free and low endotoxin; A8806) for 3 hrs. These IAV-coated plates were then incubated with SP-D preparations, followed by addition of the 246-04 monoclonal antibody (mAb) against SP-D as indicated. Secondary HRP-labeled donkey anti-mouse antibodies were then added. TMB substrate (BioRad Labs, Hercules, CA) was then added and the reaction was stopped using 1N sulfuric acid (H_2_SO_4_). The optical density was measured on an ELISA plate reader at 450 nm wavelength. Each individual data point was performed in duplicate. In some experiments SP-D was pre-incubated with mAbs (as indicated in figure 1) prior to binding to IAV.

### Hemagglutination (HA) inhibition assay

HA inhibition was measured by serially diluting collectin or other host defense protein preparations in round bottom 96 well plates (Serocluster U-Vinyl plates; Costar, Cambridge, MA) using PBS as a diluent. After adding 25 μl of IAV, giving a final concentration of 40 HA units per ml or 4 HA units/well, the IAV/protein mixture was incubated for 15 min. at room temperature, followed by addition of 50 μl of a type O human erythrocyte suspension. The minimum concentration of protein required to fully inhibit the hemagglutinating activity of the viral suspension was determined by noting the highest dilution of protein that still inhibited hemagglutination. Inhibition of HA activity in a given well is demonstrated by absence of formation of an erythrocyte pellet. If no inhibition of HA activity was observed at the highest protein concentration used then the value is expressed as > the maximal protein concentration.

### Fluorescent focus assay of IAV infectivity

MDCK monolayers were prepared in 96 well plates and grown to confluency. These layers were then infected with diluted IAV preparations for 30 min. at 37°C in PBS, followed by washing of the monolayer three times in serum free Dulbecco's Modified Eagle Medium (DMEM) containing 1% penicillin and streptomycin. The monolayers were then incubated for 7 hours at 37°C with 5% CO_2 _in DMEM. The monolayers were subsequently washed three times with PBS and fixed with 80% acetone (volume/volume) for 10 minutes at 4°C. The monolayers were then labeled by incubating with monoclonal antibody directed against the influenza A viral nucleoprotein (provided by Dr. Nancy Cox, CDC, Atlanta, GA) in reagent A (PBS with 0.1% BSA, 1% heat inactivated human serum, 0.02% NaN3) for 30 min. at 4°C. The monolayers were washed three times in PBS and incubated with FITC-labeled goat anti-mouse IgG. The fluorescent foci were counted directly under fluorescent microscopy. Initially, various dilutions of virus were used to find the dose yielding approximately 50 fluorescent foci per high powered (40×) field. These foci appeared to be single infected cells in general. In most experiments IAV was pre-incubated for 30 min. at 37°C with SP-D and/or HNPs or control buffer, followed by addition of these viral samples to the MDCK cells.

### Statistics

Statistical comparisons were made using Student's paired, two-tailed *t *test or ANOVA with post hoc test (Tukey's).

## Results

### Testing of MAbs against SP-D in viral binding and neutralization assays

In our prior report we showed that natural human SP-D multimers bind more strongly to IAV using polyclonal anti-SP-D antibodies. We wanted to confirm this finding using mAbs. In addition, we wanted to determine if mAbs alter functional interactions of SP-D with IAV. As shown in figure 1, three different mAbs were tested (245-01, 246-04 and 246-07). The 246-07 mAb inhibits binding of SP-D to mannan [31]. The 246-04 mAb inhibits binding of SP-D to gp-340 but does not inhibit binding to mannan [31]. As shown in figure 1, pre-incubation of the 246-07 mAb with SP-D strongly interfered with the IAV neutralizing activity of SP-D against the Phil82 strain of IAV. The 246-04 only slightly interfered with neutralizing activity by comparison. The 245-01 binds to the neck region of SP-D and did not interfere and at one concentration with neutralizing activity of SP-D. Similar results were obtained in HA inhibition assays: 246-07 interfered with HA inhibiting activity of SP-D but the other mAbs did not (Table 1).

**Table 1:** Effect of mAbs on ability of SP-D to inhibit viral hemagglutination activity

**MAb added:**	**None**	**245-01^a^**	**246-04**	**246-07**
HA inhibiting conc. of SP-D (ng/ml)^b^	49 ± 14	37 ± 11	55 ± 17	>420 p < 0.001

^a ^Recombinant human SP-D multimers were pre-incubated with the indicated MAbs prior to adding SP-D to virus and assaying HA inhibition.

^b ^The values shown are mean ± SEM (n = 4) amounts of SP-D needed to inhibit 40 HA units of the Phil82 strain of IAV.

We next tested the effects of the mAbs in an ELISA assay for binding of SP-D to IAV. In the first set of experiments we incubated IAV with recombinant human SP-D dodecamers and then added mAbs. Bound antibodies were detected with HRP labeled goat anti-mouse antibodies. As shown in panel B of figure 1, the 246-04 and 246-07 mAbs recognized SP-D bound to IAV to a roughly similar extent. We then tested the effect of pre-incubating SP-D with the mAbs followed by addition of the mixture to the virus-coated plates. Using this method, binding of SP-D was unaffected by the 246-04 mAb but was markedly reduced by the 246-07 mAb.

### Natural and recombinant SP-D trimers bind less strongly to IAV than multimers

**Figure 2:**
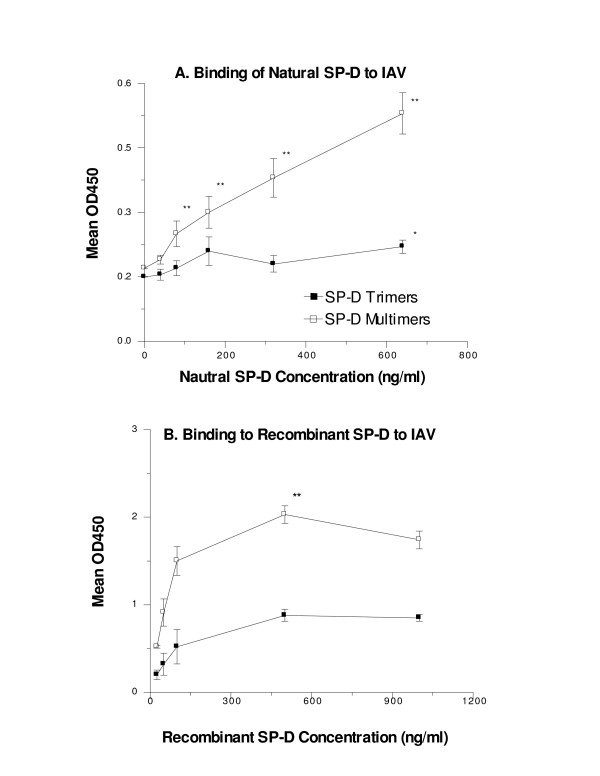
**Natural SP-D multimers bind more strongly to IAV than SP-D trimers**. Natural SP-D multimers and trimers were purified from amniotic fluid and tested for binding to Phil82 IAV by ELISA using the 246-04 mAb for detection of bound SP-D. Natural SP-D multimers showed significantly greater binding than trimers (** indicates p < 0.01; n = 3; Panel A). Binding of natural SP-D trimers was significantly above background binding to BSA at 640 ng/ml only (* indicates p < 0.01). In panel B we compared binding of recombinant human trimers and multimers isolated by gel filtration. Again binding of multimers was significantly greater than binding of trimers. In panel B background binding to BSA coated plates was subtracted from the results shown.

In initial experiments we coated natural SP-D multimers and trimers on ELISA plates and tested for detection with MAb 246-04. This mAb was found to detect natural human SP-D trimers to a similar, if not greater, extent than it detected natural SP-D multimers (data not shown). Since the 246-04 mAb also did not alter functional interactions of SP-D with IAV, we used this mAb to detect binding of the natural and recombinant human trimers and multimers to IAV (figure 2). These assays confirmed that the natural multimers bound much more strongly to IAV (figure 2; panel A). Of interest, recombinant SP-D containing the Thr11 sequence forms a complex mixture of trimers, dodecamers and high molecular weight multimers in vitro which can be separated isolated using gel filtration [9]. We compared IAV binding activity of purified trimers and high molecular weight multimers (figure 2; panel B) and found greater binding activity for the multimers using these preparations also.

### Natural human SP-D multimers have greater viral neutralizing and HA inhibiting activity than trimers

**Figure 3:**
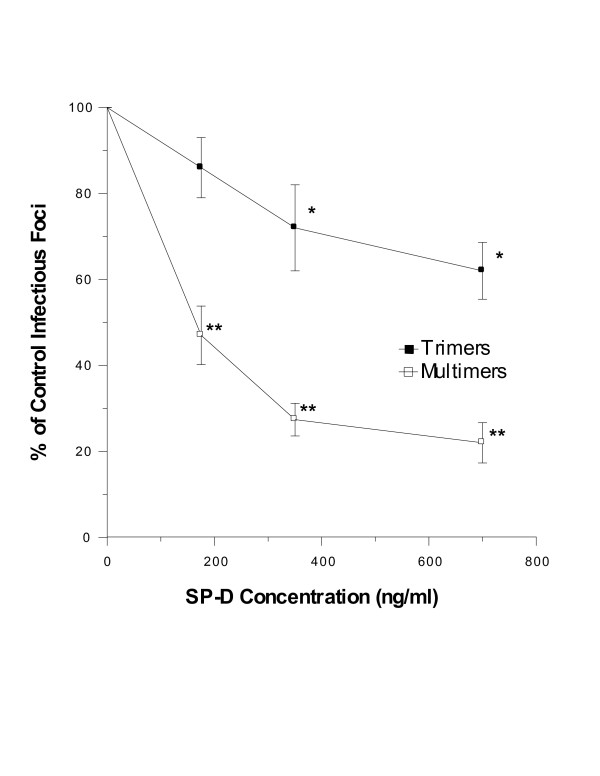
**Natural SP-D multimers cause greater neutralization of IAV than trimers**. Neutralization of IAV was assessed as in figure 1. Phil82 IAV was pre-incubated with SP-D at the indicated concentrations prior to infection of MDCK cells. Both SP-D multimers and trimers caused neutralization of IAV (* p < 0.05 compared to control); however, the effect of multimers was significantly greater than trimers (** indicates p < 0.05 compared to control or SP-D trimers). Neutralization by trimers and multimers was also significantly different when analyzed by ANOVA. Results are mean ± SEM of 5 experiments.

Using a fluorescent focus assay for viral neutralization, SP-D multimers had significantly greater neutralizing activity against the Phil82 strain of IAV compared to trimers (figure 3). Although neutralizing activity of the trimers was reduced compared to multimers, the trimers did have significant neutralizing activity. SP-D multimers also had significantly greater activity in the HA inhibition assay against three different IAV strains (Table 2). Similar results were obtained with multiple independent preparations of the natural SP-Ds (data not shown). Of interest the natural multimers had measurable HA activity against the PR-8 strain of IAV. This strain is generally resistant to collectins due to a lack of high mannose carbohydrates on its envelope proteins [7,40-42]. Activity of the SP-D multimers against PR-8 was significantly less than activity against the representative recent human strains, Phil82 and Braz79.

**Table 2:** Comparison of HA inhibition of IAV strains by SP-D multimers and trimers

**Viral Strain:**	**Phil82 H3N2**	**PR-8 H1N1**	**Braz79 H1N1**
SP-D multimers	266 ± 47^a^	2200 ± 423	259 ± 40
SP-D trimers	≥2062 ± 437 p < 0.01 vs multimers	>2500	≥1750 ± 459 p < 0.01 vs multimers

^a ^HA inhibition was measured using human Type O erythrocytes as described. The results shown are the minimal concentrations of SP-D needed to inhibit 40 HAU of the indicated viral strains (mean ± SEM; n = 3–5 experiments). Where ≥ is shown some of the values were above the maximum concentration of SP-D added (i.e., 2500 ng/ml).

### SP-D multimers have distinct interactions with HNP in viral neutralization assays

**Figure 4:**
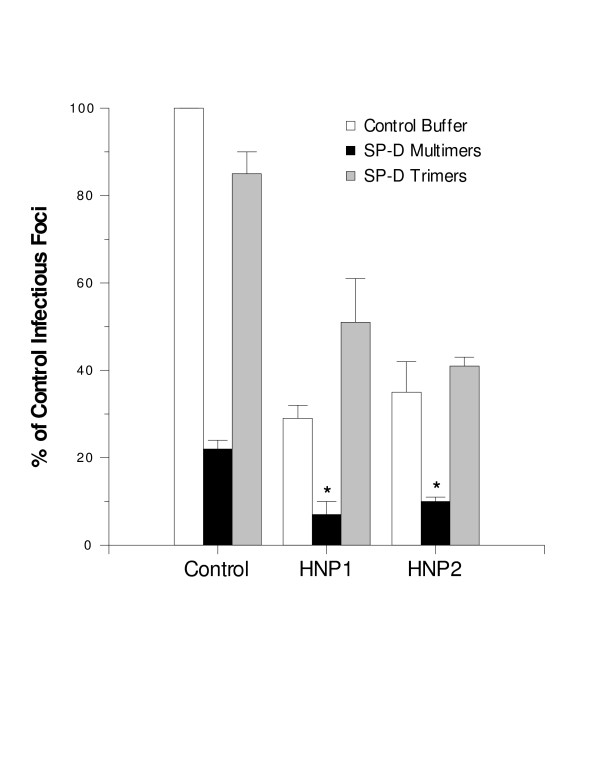
**Viral neutralization by HNPs alone or in combination with natural SP-D multimers and trimers**. Viral neutralization was assessed as in figure 1 and expressed as % of control infectious foci in samples treated with SP-D preparations alone (control; black and gray bars), HNP1 or 2 alone (HNP1 or HNP2; 5 μg/ml; white bars), or combination of HNP1 or 2 with the SP-D preparations (HNP1 or HNP2; black and gray bars; SP-D concentration 350 ng/ml). SP-D multimers caused significantly greater neutralization than trimers as in figure 3 (n = 5; p < 0.01). HNPs 1 and 2 also caused significant neutralization on their own. When SP-D multimers were combined with HNPs a significant increase in neutralization was observed (* indicates significantly different from control or SP-D multimers or HNPs alone by ANOVA).

We have previously reported that SP-D binds to HNPs via its CRD through a mechanism that is not dependent on calcium or saccharides, but increased at low pH and low ionic strength [28]. As in the case of gp-340, complex functional interactions between HNPs and SP-D were observed in antiviral assays. In some cases HNPs inhibited antiviral activity of SP-D (and vice versa); less often additive effects were observed. Of interest, we found that natural human SP-D multimers and trimers had equivalent binding to HNPs [28]. As shown in figure 4, HNPs 1 and 2 had strong viral neutralizing activity against the Phil82 strain of IAV. Natural SP-D multimers had additive effects when combined with HNP1 or HNP2 in these assays. As in figure 3, SP-D multimers had greater independent neutralizing activity than trimers. SP-D trimers did not have additive effects with the HNPs, rather they showed a trend toward interference with the antiviral activity of HNPs.

## Discussion

There is increasing evidence that the relatively common Thr/Thr11 polymorphism of SP-D has important implications for innate host defense against infection. This polymorphism is associated with reduced serum levels of SP-D and generation of predominantly trimeric SP-D *in vitro *and *in vivo *[34]. Furthermore the Thr/Thr11 polymorphism has been associated with increased risk for *M. tuberculosis *in Mexico [36]. We now confirm that natural SP-D trimers have reduced ability to bind to IAV, and further demonstrate that the trimers have reduced neutralizing and HA inhibiting activity against IAV. It will be important to confirm these results with more preparations of natural SP-D, including samples obtained from bronchoalveolar lavage fluid, and recombinant forms of the Thr/Thr11 and Met/Met11 variants; however, the findings are consistent with prior studies using other full length trimeric and more highly multimerized forms of recombinant SP-D [9,33,43]. The difference in activity appears to relate predominantly to differences in multimerization since trimers and multimers of recombinant SP-D (both of which were derived from a plasmid containing the Thr11 sequence) also showed differences in viral binding activity. Recent studies have also shown that a recombinant mutant form of SP-D that lacks the collagen domain altogether but can still form multimers by disulfide bond formation at the N-terminus retains strong antiviral activity in vitro and in vivo [44]. This finding strongly supports the concept that multimerization is a major determinant of antiviral activity.

An unresolved question is why subjects with the Thr11/Thr11 genotype tend to predominantly form trimers and to have lower blood levels of SP-D in vivo, whereas recombinant proteins with this genotype can form dodecamers and multimers as well as trimers. It is possible that the Thr11/Thr11 variant, or multimers formed by it, are less stable in vivo for reasons yet to be determined.

Our findings obtained with mAbs directed against SP-D are of independent interest, since they show that an antibody directed against the region of the SP-D CRD that mediates binding to gp-340 does not interfere with antiviral activities of SP-D. Furthermore, we confirm that the carbohydrate binding activity of SP-D (as assessed with the 246-07 mAb that blocks SP-D binding to mannan) is critical for antiviral activity. Further studies of epitope mapping of the SP-D CRD coupled with studies on the ability of mAbs to interfere with functional activities will be of interest.

Overall our findings suggest that the Thr/Thr11 variant may be associated with increased susceptibility to IAV infection and that studies correlating SP-D genotype with IAV susceptibility should be pursued. Since SP-D is one of a number of innate factors involved in defense against IAV, such studies may be most fruitfully carried out in high risk populations (e.g., the elderly, infants, or subjects with other impairments of host defense) where effects of SP-D would be more likely to manifest themselves. The trimers also had different interactions with HNPs. The findings could have implications for host defense in some circumstances (e.g., inflammatory conditions where high concentrations of HNPs are present in the lung).

It is unclear what evolutionary pressure may have led to retention of an apparently less active form of SP-D. Mannose-binding lectin (MBL) deficiency is also present in a percentage of many populations and is associated with increased severity of a variety of infections. However, MBL deficiency also seems to play a deleterious pro-inflammatory role in some circumstances and subjects with MBL deficiency are protected against leishmaniasis and *Mycobacterium tuberculosis *associated meningitis [45]. One possibility, therefore, is that the Thr/Thr11 or other common polymorphic forms of SP-D are associated with reduced morbidity for some infections. SP-D plays an important role in innate defense against RSV infection based on *in vitro *and murine *in vivo *studies [46]. It is interesting therefore that the Met/Met11 polymorphism is associated with more severe RSV infection [37]. We speculate that this may reflect increased inflammatory responses to RSV among subjects with higher levels and/or predominantly multimeric SP-D. The only basis for this speculation is that we found that natural SP-D trimers caused less potentiation of neutrophil respiratory burst responses to IAV than multimers [32]. It may be, therefore, that despite reduced neutralizing activity of SP-D trimers, they are less likely to promote inflammatory responses that could be harmful. Findings of Gardai et al suggest that SP-D may play either pro- or anti-inflammatory roles depending on whether it binds to macrophages via its CRD or its collagen domain [47]. These domains have distinct receptors on macrophages which mediate anti- or pro-inflammatory effects, respectively. It is unclear how oligomerization of SP-D impacts on these interactions.

Studies in SP-D knockout mice show that the absence of SP-D is associated with increased replication of IAV and RSV and increased inflammatory responses and illness [12,48]. In the case of RSV instillation of trimeric neck and CR domain fragments of SP-D reduces severity of infection in mice [49]. In the case of IAV over-expression of a mutant trimeric form of SP-D in the lung of SP-D -/- mice reduces both viral titers and inflammatory responses without correcting any of the baseline abnormalities of the mice [50]. Hence, it is possible that the neutralizing activity of SP-D trimers is sufficient to contain RSV or IAV infection. Whether trimeric SP-D holds any advantage with regard to inflammatory responses is not clear as yet. Hopefully epidemiological studies will clarify the role of SP-D oligomerization with respect to various infections like IAV. Another important clinical study will be to determine the impact of the Thr/Thr 11 polymorphisms on levels of SP-D in BAL fluid, since this also could have an impact on host defense.

## Conclusion

Polymorphic variations in SP-D result in differences in serum levels and oligomeric structure of the protein. Most importantly, the common Thr/Thr11 polymorphism is associated with assembly predominantly as trimers, whereas the more common forms of SP-D are assembled into dodecamers or higher molecular weight multimers. Natural trimeric SP-D has reduced ability to bind to and neutralize IAV as well as distinct interactions with HNPs as compared to natural SPD multimers. These findings suggest that polymorphisms of SP-D could contribute to susceptibility of certain individuals to IAV infection. In addition, mAbs directed against different areas on the CRD of SP-D have differing effects on antiviral activity and may be useful in future studies to dissect out, or interfere with, various functional properties of SP-D.

## Competing interests

The author(s) declare that they have no competing interests.

## Authors' contributions

KLH planned experiments and prepared the manuscript.

MRW carried out viral preparation and most of the experiments involving viral binding and inhibition and reviewed manuscript.

TT assisted in viral preparation and in some of the viral binding assays and reviewed the manuscript.

IT purified SP-D fractions from amniotic fluid and in preparation of mAbs and reviewed the manuscript.

GLS assisted in purification of SP-D fractions from amniotic fluid and in preparation of mAbs and reviewed the manuscript.

ECC prepared recombinant human SP-D and polyclonal antibodies and participated in developing experimental plans and revision of manuscript.

UH originally developed mAbs to SP-D and the techniques involved in purification of SP-D fractions from amniotic fluid and participated in developing experimental plans and revision of manuscript.
